# *Escherichia coli* redox mutants as microbial cell factories for the synthesis of reduced biochemicals

**DOI:** 10.5936/csbj.201210019

**Published:** 2013-01-18

**Authors:** Jimena A. Ruiz, Alejandra de Almeida, Manuel S. Godoy, Mariela P. Mezzina, Gonzalo N. Bidart, Beatriz S. Méndez, M. Julia Pettinari, Pablo I. Nikel

**Affiliations:** a Departamento de Química Biológica (IQUIBICEN-CONICET), Facultad de Ciencias Exactas y Naturales, Universidad de Buenos Aires, Buenos Aires, Argentina; b Instituto de Biociencias Agrícolas y Ambientales (INBA-CONICET), Facultad de Agronomía, Universidad de Buenos Aires, Buenos Aires, Argentina; c Instituto de Investigaciones Biotecnológicas “Dr. Rodolfo A. Ugalde” (IIB-CONICET), Universidad Nacional de San Martín, San Martín, Buenos Aires, Argentina

**Keywords:** Escherichia coli, ArcBA, CreBC, reduced biochemicals, redox homeostasis, polyhydroxyalkanoates, metabolic flux analysis

## Abstract

Bioprocesses conducted under conditions with restricted O_2_ supply are increasingly exploited for the synthesis of reduced biochemicals using different biocatalysts. The model facultative aerobe *Escherichia coli*, the microbial cell factory *par excellence*, has elaborate sensing and signal transduction mechanisms that respond to the availability of electron acceptors and alternative carbon sources in the surrounding environment. In particular, the ArcBA and CreBC two-component signal transduction systems are largely responsible for the metabolic regulation of redox control in response to O_2_ availability and carbon source utilization, respectively. Significant advances in the understanding of the biochemical, genetic, and physiological duties of these regulatory systems have been achieved in recent years. This situation allowed to rationally-design novel engineering approaches that ensure optimal carbon and energy flows within central metabolism, as well as to manipulate redox homeostasis, in order to optimize the production of industrially-relevant metabolites. In particular, metabolic flux analysis provided new clues to understand the metabolic regulation mediated by the ArcBA and CreBC systems. Genetic manipulation of these regulators proved useful for designing microbial cells factories tailored for the synthesis of reduced biochemicals with added value, such as poly(3-hydroxybutyrate), under conditions with restricted O_2_ supply. This network-wide strategy is in contrast with traditional metabolic engineering approaches, that entail direct modification of the pathway(s) at stake, and opens new avenues for the targeted modulation of central catabolic pathways at the transcriptional level.

## INTRODUCTION

Anoxic fermentation of different carbon sources is rapidly gaining momentum in biotechnological setups aimed at obtaining reduced biochemicals. Relevant examples in this sense include (but are certainly not limited to) the production of polyhydroxyalkanoates (PHAs) [1, 2], ethanol [3, 4], 1,3-propanediol (1,3-PDO) [5, 6], succinate [7], and D-lactate [8]. These reduced biochemicals possess great commercial interest, and are usually synthesized by different microorganisms through the activity of both native and heterologous pathways. In spite of the fact that several bacterial species are currently being used in biotechnological setups, *E*. *coli* remains as the microbial cell factory (MCF) *par excellence*, mainly because this well- characterized enterobacterium can easily and rapidly grow on cheap substrates and can be modified as desired through a broad variety of molecular tools. As fermentation technologies designed for the production of bulk bioproducts need further innovation to became economically and environmentally sound [9], there is an increasing interest in methodologies that optimize the production of biochemicals, such as those listed above, according to both market demands and bioprocess energy requirements. It has been proposed that the most probable avenue for future improvements of these strategies will rely on a combination of efficient fermentation processes and in the manipulation of the MCF metabolism employing metabolic engineering strategies.

Metabolic manipulations to enhance the synthesis of metabolic products include several approaches to increase the availability of substrates needed for its formation and/or to eliminate competing pathways that lead to the formation of by-products, which sometimes conduces to undesired phenotypes. An alternative strategy that has been scarcely exploited for the design and optimization of MCFs is the network-wide manipulation of metabolic fluxes by means of mutations in global regulators. In fact, and depending on environmental circumstances, this approach can outperform the more traditional metabolic engineering strategy based on the direct manipulation of the gene(s) involved in the pathway(s) of interest. In the present mini-review, we summarize the recent advances and current state on the use of redox and/or regulatory *E*. *coli* mutants as MCFs for the production of reduced biochemicals and recombinant proteins. In the first part, we present some general aspects of the microbial metabolism that are subjected to control by environmental conditions through the regulation exerted by two-component signal transduction systems, namely ArcBA and CreBC. The application of targeted mutants in these regulatory systems in processes aimed at the synthesis of PHAs, under conditions with restricted O_2_ supply, is then presented along with other examples of products with commercial value. Future directions for improvement of these redox MCFs are finally discussed under the light of synthetic biology, computer-aided modeling, and other *in silico* strategies.

## REGULATION OF CENTRAL METABOLIC PATHWAYS IN *E*. *coli* BY ENVIRONMENTAL CONDITIONS: NEW LESSONS FOR AN OLD HISTORY

**Figure 1:**
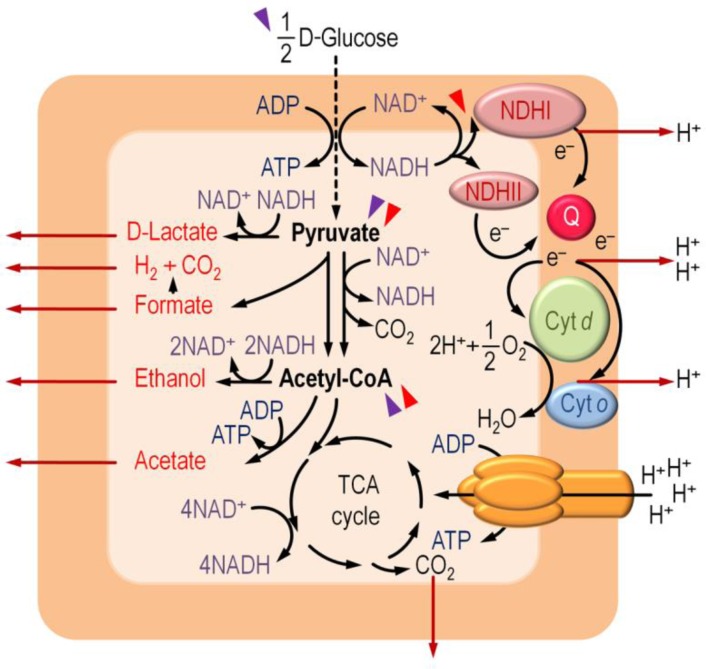
Simplified representation of the oxic and anoxic pathways for D-glucose catabolism in *E*. *coli*. Oxic pathways are sketched to the right of the outline, and anoxic pathways are represented to the left along with the main fermentation metabolites formed (in red). The initial catabolic steps of D-glucose through the Embden-Meyerhof-Parnas pathway, which are independent of the presence of O_2_, are indicated by a dashed vertical arrow. Central metabolites relevant for the production of the reduced biochemicals discussed in the text are shown in boldface. The double arrow representing the conversion of pyruvate into acetyl-CoA illustrate the activity of either the pyruvate dehydrogenase complex (mostly under oxic conditions, right), or pyruvate-formate lyase (mostly under anoxic conditions, left). Secretion of fermentation metabolites and active H^+^ pumping are indicated by red arrows. Slanted arrowheads identify metabolic steps that are subjected to regulation by the ArcBA system (red) and/or the CreBC system (purple). Abbreviations are as follows: NDHI and NDHII, NADH:ubiquinone oxido-reductases I and II, respectively; Q, (ubi)quinone/(ubi)quinol; Cyt *d* and Cyt *o*; cytochromes *d* and *o* oxidases, respectively; CoA, coenzyme A; TCA cycle, tricarboxylic acid cycle. *Nota bene*, although the TCA cycle is depicted as an entirely oxic sequence of reactions in this scheme, the reductive branch, active under conditions with restricted O_2_ supply, produces succinate as a fermentation metabolite.

As metabolic engineering approaches become more and more complex in the pursuit of the ideal MCF for the production of reduced biochemicals, the need of a complete understanding of cell physiology and metabolic network operativity under micro-oxic and anoxic growth conditions also becomes apparent. Recent genome-wide models revealed new aspects of the regulation of these pathways, which have a deep impact in the energy and redox homeostasis of the cells and, consequently, on the strategies implemented in order to manipulate these traits. A brief summary of the core *E*. *coli* metabolism is presented below, and the different pathways for D-glucose utilization are depicted in Fig. 1 along with some key regulatory checkpoints within the native metabolic network.

The initial steps in the catabolism of D-glucose, the preferred carbon source of *E*. *coli* and the substrate most often used in biotechnological setups, are independent of O_2_ availability. D-Glucose is split into the Embden-Meyerhof-Parnas (EMP) pathway and the pentose phosphate (PP) pathway at the D-glucose-6-*P* branching point [10]. The oxidation of D-glucose-6-*P* to D-ribose-5-*P via* the PP pathway generates reducing equivalents (*i*.*e*., NADPH) that are mostly used for biosynthesis and to quell stressful conditions. Degradation of D-glucose-6-*P* by the EMP pathway, in contrast, generates pyruvate, which under oxic conditions is decarboxylated by the pyruvate dehydrogenase complex to produce acetyl-coenzyme A (CoA), NADH, and CO_2_ [11, 12]. Oxidation of acetyl-CoA in the tricarboxylic acid (TCA) cycle generates electron carriers (*i*.*e*., NADH and FADH_2_), channeled into the electron-transport chain to finally reduce O_2_ to H_2_O, creating a H^+^ gradient used to generate ATP by means of ATP synthase. D-Glucose is completely oxidized to CO_2_ and H_2_O to conserve energy during oxic respiration, thus giving the maximum ATP yield.

When O_2_ is not available, *E*. *coli* undergoes anoxic respiration as long as an alternative electron acceptor is present in the surrounding environment [13]. Electron-transport chains of *E*. *coli* comprise dehydrogenases and terminal oxidases linked to a complex (ubi)quinone pool [14]. The presence of a particular electron acceptor under anoxic conditions results in the transcriptional activation of specific oxido-reductases and dehydrogenases and, concomitantly, in the repression of alternative oxido-reductases [14, 15]. This transcriptional control is exerted by global regulatory proteins (*e*.*g*., Fnr, ArcA, NarQ, NarX), which respond to changing levels of electron acceptors (O_2_ in the case of Fnr and ArcA, and nitrate in the case of NarQ and NarX) to maximize metabolic efficiency [10, 14–17], see also next section).

In the absence of any terminal acceptor, substrate-level phosphorylation is used for energy production and redox homeostasis is achieved by transferring electrons from reducing equivalents to an internal electron acceptor, such as pyruvate or acetyl-CoA [18]. Mixed acid fermentation [19, 20] thereby generates the overflow metabolites acetate, ethanol, formate, D-lactate, and succinate. Under anoxic conditions, pyruvate generated through the EMP pathway is processed by the radical enzyme pyruvate formate-lyase (PFL), leading to the production of acetyl-CoA and formate (with a lesser contribution of the mostly aerobic pyruvate-dehydrogenase complex [21, 22]). Expression of the *pfl* genes is controlled by several transcription factors [23–28], and PFL is mainly active in the absence of O_2_ [29], although it can contribute to acetyl-CoA formation under micro-oxic conditions [30, 31]. Formate produced by PFL can be converted to H_2_ and CO_2_ by formate-hydrogen lyase [32–34]. On the other hand, acetyl-CoA produced by PFL can be converted into ethanol or acetate. Reduction of acetyl-CoA to ethanol is catalyzed by an acetaldehyde/alcohol dehydrogenase (AdhE), and production of acetate from acetyl-CoA generates ATP by substrate-level phosphorylation [35]. This two-step reaction is catalyzed by the sequential action of the enzymes phosphotransacetylase (Pta) and acetate kinase (AckA), encoded by the *ackA*-*pta* operon. The metabolic fate of acetyl-CoA largely depends on the amount and type of the carbon source used and the availability of reducing equivalents. As mentioned above, minor amounts of D-lactate and succinate are also produced during mixed acid fermentation. D-Lactate is formed by the reduction of pyruvate by the NADH-dependent D-lactate dehydrogenase (LdhA) [36]. Succinate is produced by the reductive decarboxylation of *P*-*enol*-pyruvate to fumarate by fumarate reductase, as a result of the repression of the oxidative branch in the TCA cycle [37, 38]. The presence of alternative carbon sources in the culture medium also alters the pattern of metabolic pathways active within the cell. Although not extensively considered in the present mini-review, some interesting approaches designed for metabolic engineering applications involve the use of glycerol (see next sections), pentoses, and short-chain fatty acids [39–43].

All the above mentioned metabolic pathways are ultimately regulated by the availability of O_2_, and the delicate redox and energy balances are tightly controlled at several levels. On top of them all, transcriptional regulation constitutes the predominant mechanism whereby the activity of the relevant pathway(s) is temporally and spatially coordinated in order to meet homeostasis. The next section describes the main patterns of global regulation that ultimately ensure the network-wide adjustment of metabolic pathway activity.

## REGULATORY TASKS OF THE ArcBA AND CreBC TWO-COMPONENT SYSTEMS ON THE CENTRAL METABOLIC PATHWAYS OF *E*. *coli*

Signal transduction pathways are involved in intercellular interactions and communication of environmental conditions to the interior of the cell. The final outcome of such a signaling pathway is the activation of specific transcription factor(s) that, in turn, control(s) gene expression. Regulation of gene expression is a very complex process, and transcriptional regulators can be subdivided in global and local regulators depending on the number of operons (*i*.*e*., regulons) they control. Global regulators control the expression of a vast number of genes, which might be physically scattered along the genome and normally belong to different functional clusters. According to the data available in the EcoCyc database [44], *E*. *coli* K12 strain MG1655 contains forty master (*i*.*e*., global) regulators and σ factors. Perhaps surprisingly, only seven global regulators control the expression of *ca*. half of all genes; *i*.*e*., ArcA, Crp, Fis, Fnr, Ihf, Lrp, and NarL [45]. In contrast to these global regulators, local regulators control the expression of only a few genes, and *ca*. one-fifth of all the transcriptional regulators so far described control the expression of just one or two genes.

O_2_ functions as an electron acceptor and substrate for catabolism in a wide variety of bacteria, and most facultative anaerobe microorganisms are able to thrive across a wide range of O_2_ availability conditions. As hyperoxic conditions may produce adverse effects in bacteria by inducing oxidative stress, micro-aerobic and facultative anaerobic bacteria have evolved elaborate sensing and signal transduction mechanisms in order to adapt their metabolism in response to O_2_ availability [46]. As explained before, the choice of energy generation pathways is determined by the accessibility to electron acceptors. Sophisticated and often interrelated regulatory networks switch the expression of these pathways on and off as needed [13, 15]. In the hierarchical regulation system for energy transduction, adaptive responses to O_2_ are mainly coordinated by the Fnr and ArcA global regulators [47]. The transcriptional regulation exerted by Fnr is mostly related to strict anoxic conditions, and its effects have been reviewed elsewhere [48, 49].

The ArcBA (anoxic redox control) two-component signal transduction system modulates at the transcriptional level the expression of many operons according to the redox state of the environment [50–53]. ArcB is a transmembrane sensor kinase which under anoxic or micro-oxic conditions undergoes stable phosphorylation and then transphosphorylates the response regulator ArcA. The main targets for repression by ArcA∼*P* are the genes that encode enzymes involved in oxic respiration. On the other hand, the cytochrome *d* oxidase, with high O_2_ affinity, and genes encoding fermentation enzymes such as PFL, are activated under anoxic conditions. The effects of ArcA∼*P* on the transcription patterns has been analyzed at the whole-genome level, and it was shown that many other genes, far beyond those directly involved in redox metabolism, are putative targets for ArcA regulation. In fact, it was found that *ca*. 150 operons involved in energy metabolism, nutrients transport, bacterial survival, catabolism of diverse carbon sources, and transcriptional regulation, were included in the Arc modulon. Later on, statistical analysis of genome-wide DNA microarrays led to the striking conclusion that the transcription of at least 1,139 genes of *E*. *coli* could be either directly or indirectly regulated by ArcA∼*P* [54].

CreBC (carbon source responsive) is also a global sensing and regulation system responsive to both the carbon source and the O_2_ availability [55]. The *cre* locus comprises *creABCD* and was formerly known as *phoM* locus. While *creA* is a hypothetical open reading frame and *creD*, also known as *cet*, encodes an inner-membrane protein of unknown function; *creB* and *creC* encode a two-component system, *i*.*e*., a cytoplasmic regulator and a sensor kinase, respectively. Following the discovery of CreBC and its recognition as a sensing/regulatory pair, genes modulated *bona fide* by this system proved very elusive. Based on genome-wide sequence analysis and using bioinformatic tools, Avison *et al*. [55] were able to define a so-called *cre* tag sequence, to which CreB is known to bind *in vitro*, in order to describe the Cre regulon. So far, the Cre regulon comprises seven genes activated by CreBC (among them, *ackA*-*pta*, *talA*, and *creD*) and one repressed (*malE*), and the expression pattern of these genes responds to the nature of the carbon source used to grow the cells and the O_2_ availability. Indeed, expression of genes modulated by CreBC is activated under fermentative growth conditions using glycolytic carbon sources, as well as under oxic conditions with low-molecular-weight fermentation products as the substrate, such as formate or pyruvate. Yet, very little is known about this system and the extent of its regulation on the central metabolism of *E*. *coli*.

The intracellular distribution of metabolites (and, consequently, the phenotypic traits of the cell) is regulated at several levels. Transcriptional regulation is thought to be the principal type of regulation in bacteria, yet the way in which it controls metabolic fluxes is not well understood. Several studies have recently tried to clarify the correlation between metabolic networks and transcriptional control by means of metabolic flux analysis in global regulatory mutants. A breakthrough in fluxome quantification strategies was the use of ^13^C-substrates, which proceed through the entire metabolic network and propagate their labeling pattern into the pools of downstream metabolites, thus allowing to calculate the flux(es) through the pathway(s) at stake [56–59]. Since ArcA has been identified as a major global regulator, several works were dedicated to unravel its role in controlling the *E*. *coli* central metabolism. One of them dealt with different global regulatory mutants of *E*. *coli* grown on D-glucose batch cultures [60]. This study provided novel insights into the regulation brought about by the ArcBA system, demonstrating that, unexpectedly, the control of fully oxic and anoxic fluxes through the TCA cycle was exerted by ArcA in an ArcB-independent fashion. ^13^C-labeling experiments were also performed in ∆*arcA* and ∆*fnr* mutants in D-glucose − limited chemostat cultures [61]. These mutations were shown to affect the flux distribution in the core metabolic pathways, mainly through the availability of reducing equivalents.

As both ArcBA and CreBC regulate the expression of genes encoding enzymes of the central catabolic pathways (see Supplementary Table S1 for details), the next relevant question was whether there is a shared control of metabolism by the two systems. ^13^C-Based metabolic flux analysis conducted for a wild-type *E. coli* strain and its isogenic ∆*creB*, ∆*arcA*, and ∆*creB* ∆*arcA* derivatives in D-glucose- and O_2_-limited chemostat experiments demonstrated that both ArcBA and CreBC exert an important influence on central carbon catabolic fluxes [62]. In particular, it was observed that both the *P*-*enol*-pyruvate and the acetyl-CoA metabolic nodes are subjected to regulation by CreB and ArcA, strongly influencing the fate of the carbon atoms at these branching points. Interestingly, the first steps on D-glucose catabolism did not seem to be affected, and most of the carbon source was channeled through the EMP pathway without evident differences between the wild-type strain and its mutant derivatives.

At this point, it is relevant to consider that the genotype-phenotype relationships in regulatory mutants are usually very complex, and the deletion of global regulators may have an impact beyond the redox and catabolic traits of the cells. The combined approaches discussed in this section for the analysis of regulatory mutants are relevant since deletion of global regulatory genes is expected to affect the entire cellular and metabolic landscape in a rather difficult-to-predict fashion. Moreover, possible cross-talk mechanisms between these two-component systems [63, 64] could also contribute to the complex biochemical signalization in the resulting MCFs, and modulation of the Arc and/or Cre signalization by different levels of fermentation metabolites [65] cannot be ruled out. One way or the other, the results of the studies discussed above underscored the potential of *arc* and *cre* mutants as MCFs, and they were utilized for the production of different reduced biochemicals, as detailed in the next sections.

## BACTERIAL POLYHYDROXYALKANOATES

**Figure 2:**
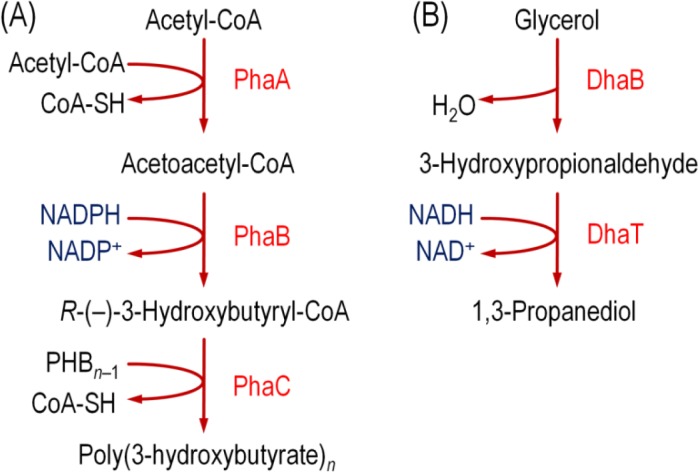
Biosynthetic pathways of two model reduced biochemicals, poly(3-hydroxybutyrate) and 1,3-propanediol. Biochemical steps that consume reducing equivalents are highlighted in blue. Abbreviations are as follows: CoA, coenzyme A; PhaA, 3-ketoacyl-CoA thiolase; PhaB, acetoacetyl-CoA reductase; PhaC, poly(3-hydroxyalkanoate) synthase; DhaB, glycerol dehydratase; DhaT, 1,3-propanediol oxidoreductase. In most of the *E*. *coli* recombinants described in the text, the *phaBAC* genes were obtained from *Azotobacter* sp. strain FA8 [70].

PHAs are synthesized naturally by a wide variety of bacterial species as a reserve material for carbon and energy [2, 66]. These ubiquitous polymers attract increasing industrial interest as renewable, biodegradable, biocompatible, and extremely versatile thermoplastics [2]. In fact, PHAs are the only H_2_O-proof thermoplastic materials which are also fully biodegradable in both oxic and anoxic environments. Two classes of PHAs are distinguished according to their monomer composition: short-chain length (SCL) PHAs, and medium-chain length (MCL) PHAs. SCL-PHAs are composed of 3-hydroxyacid monomers with a chain length of three to five carbon atoms, such as poly(3-hydroxybutyrate) (PHB, the most common and widely distributed PHA in nature); whereas MCL-PHAs contain 3-hydroxyacid monomers with six to sixteen carbon atoms. All of these polymers are optically active *R*-(—)-compounds, and give rise to isotactic carbon chains. The structural versatility of PHAs is partly due to the wide substrate range of the polymer-synthesizing enzymes, and it endows PHAs with an extended spectrum of mechanical and physical properties, a clear advantage *vis-à-vis* to other bioplastics. More than 200 different monomer constituents have been found so far in these polymers [67]. PHB is synthesized from acetyl-CoA in a three-step pathway (Fig. 2A), that uses NADPH as cofactor.

The design and development of efficient MCFs for PHA production received much attention in the last few decades. Although different microorganisms have been exploited for polymer production, *E*. *coli* is most often used as the biocatalyst [68] as it does not accumulate PHAs naturally, thus offering the flexibility to manipulate both native and heterologous pathways for PHA synthesis. Current high-yield bioprocesses for the synthesis of PHAs require fully oxic conditions, meaning that they are high energy-consuming processes. The environmental impact of replacing oil-derived plastics with biopolymers has been the subject of several studies, among them, those regarding technical-scale PHB production in bioreactors [69]. The main conclusion of these studies is that when the total amount of energy invested is taken into consideration [*i*.*e*., the energy needed for sterilization, aeration, and agitation (both in the bioreactor and downstream processing), as well as the energy for the production of agricultural feed-stocks used as carbon sources], the environmental performance of PHAs equals that of petrochemical polymers. This situation clearly calls for alternative bioprocesses and biocatalysts to meet the technical and environmental requirements of a sound production process.

Metabolic networks are the most obvious targets for rational design of sustainable PHA production processes. As stated above, *E*. *coli arc* mutants are unregulated for respiration under micro-oxic conditions. Some enzymes of the TCA cycle are not repressed and the pool of reducing equivalents is elevated, and thus available to be funneled into reduced biochemicals. The PHB biosynthetic genes from *Azotobacter* sp. strain FA8 [70] were introduced in a Δ*arcA* strain, and the polymer accumulation was evaluated under micro-oxic conditions in a semi-synthetic medium containing D-glucose as the carbon source. A PHB content of *ca*. 35% (w/w) was attained in such a redox mutant, while the wild-type strain failed to produce PHB under the same culture conditions [71]. Within the same conceptual framework, PHA synthesis was evaluated in *E*. *coli* CT1061, an *arcA* and *creC* constitutive mutant. The latter mutation confers enhanced carbon source consumption, while retaining the markedly reducing intracellular environment typical of *arc* mutants (with a NADH/NAD^+^ ratio of *ca*. 1 mol/mol) [72]. *E*. *coli* CT1061 actually outperformed *cre* and *arc* individual mutants as a redox MCF. Increased PHB yields on substrate were observed in D-glucose- or glycerol-supplemented semi-synthetic media [73], especially in micro-oxic fed-batch cultures with glycerol supplementation. Being the main by-product of the biodiesel industry, glycerol became a cheap substrate for bioprocesses in the last few years. Since C atoms are in a more reduced state than those in hexoses, the use of glycerol as a biotechnological substrate is especially attractive in bioprocesses designed to produce reduced biochemicals [74, 75]. The micro-oxic fed-batch cultures afore mentioned allowed a 2.6-fold increase in the PHB volumetric productivity when compared to batch cultures. After 60 h, biomass and PHB concentrations reached 21.17 and 10.81 g/L, respectively, resulting in a PHB content of 51% (w/w) [76]. Ethanol was co-produced as a valuable by-product of this process [77], making it even more interesting in terms of sustainability, as two industrially-relevant reduced biochemicals were obtained from glycerol, both intracellularly (PHB) and extracellularly (ethanol).

The main goal of a successful heterologous expression system based on plasmids is to achieve the highest tolerable protein or metabolite yield and thus to walk the thin line between high levels of heterologous gene expression and the metabolic capacity of the host. An unexpected (and most welcome) trait of MCFs bearing *arc* and *cre* mutations is an enhanced ability to maintain plasmids that encode the biosynthetic genes for reduced biochemicals (*e*.*g*., ethanol and PHB), even in the absence of the selective pressure imposed by addition of antibiotics [78]. This side effect was shown to be mostly related to the capacity enabled by the plasmid-encoded bioreactions to regenerate NAD(P)^+^, needed to continue cell growth.

Another target of global regulation exploited for the heterologous accumulation of PHB in *E*. *coli* is the AtoSC two-component system, which regulates the catabolism of short-chain fatty acids. Theodorou *et al*. [79] recently demonstrated that PHB synthesis from D-glucose is impaired in a Δ*atoSC* and Δ*atoDAEB* mutant, and that this phenotype can be restored by ectopic expression of Ato components in the recombinants. The authors hinted an important role of the AtoSC system in polymer accumulation and also proposed that this regulatory system could be an important target for metabolic engineering manipulations. In a former study, Pettinari *et al*. [80] proposed the use of a transcriptional activator of the *ato* genes from *Bacillus megaterium* as an elegant strategy to obtain different types of PHAs in recombinant *E*. *coli* MCFs.

A different approach was the use of anoxic promoters to drive the transcription of the *pha* genes from *Cupriavidus necator* in *E*. *coli* recombinants under conditions with restricted O_2_ supply [81]. Among the nine native promoters tested, P_*adhE*_ was the most effective in promoting micro-oxic PHB synthesis. The same research group recently reported the construction of an *E*. *coli* recombinant that carries the *pha* genes from *C*. *necator*, as well as the genes encoding hydrogenase 3 and/or acetyl-CoA synthetase [82]. The resulting strain had the advantage of co-producing PHB and H_2_ under micro-oxic conditions in a synthetic medium containing D-glucose and acetate as the carbon sources.

## 1,3-PROPANEDIOL

1,3-PDO is used in many synthetic reactions, particularly as a monomer for condensations to produce polyesters, polyethers, and polyurethanes [83]. Polymers made with this compound are biodegradable and have enhanced chemical properties, such as higher light stability and solubility [6]. 1,3-PDO is primarily produced through chemical synthesis from petroleum derivatives (acrolein and ethylene) in processes that involve high pressure and temperature, but it can also be obtained by microbial fermentation, a more environmentally-favorable process that has lower costs, especially considering its production from glycerol, the abundant by-product of the biodiesel synthesis [84]. 1,3-PDO was originally identified by August Freund in a glycerol fermentation process with a mixed culture containing *Clostridium pasteurianum* [85], and was later found to be produced by the fermentation of glycerol by many Gram-positive and Gram-negative bacteria, including *Citrobacter*, *Clostridium*, *Enterobacter*, *Klebsiella*, and *Lactobacillus* species [86]. As the anoxic growth on glycerol generates an excess of reducing equivalents, it causes a redox imbalance. In order to meet redox homeostasis, cells synthesize products that serve as an electron sink, such as 1,3-PDO. This molecule is synthesized in two steps: [i] dehydrogenation of glycerol to 3-hydroxypropionaldehyde, and [ii] an NADH-dependent reduction of this intermediate to 1,3-PDO, that is secreted into the medium [86, 87] (Fig. 2B). In microorganisms that produce 1,3-PDO naturally, the enzymes involved in glycerol metabolism are a glycerol dehydratase, a 1,3-PDO oxidoreductase, a glycerol dehydrogenase, and a dihydroxyacetone phosphate kinase. These enzymes are encoded in the *dha* operon, which has been characterized in *K*. *pneumoniae*, *C*. *freundii*, and *C*. *butyricum* [6].

Many natural 1,3-PDO producers are not suitable for industrial production because of their particular culture conditions and, in the case of pathogens, because special safety precautions are required [5]. For these reasons, there has been recent interest in converting glycerol to 1,3-PDO by recombinant *E*. *coli* strains. Such MCFs also permit the study of the effects of different mutations in genes involved in metabolism and global regulators in order to improve the synthesis of 1,3-PDO. Several research groups have reconstructed the 1,3-PDO pathway in *E*. *coli*, normally containing genes from *K*. *pneumoniae* or *C*. *butyricum* [88, 89]. The highest 1,3-PDO production yield with glycerol as the sole carbon source reported so far was in a recombinant *E*. *coli* strain expressing *dhaB1* and *dhaB2* from *C*. *butyricum*, which encode the vitamin B_12_-independent glycerol dehydratase DhaB1 and its activating factor, DhaB2, respectively, tandemly arrayed with *yqhD* from *E*. *coli*, which encodes the 1,3-PDO oxidoreductase isoenzyme YqhD, an NADPH-dependent dehydrogenase [90].

Generally, it can be considered that availability of NADH is one of the main factors limiting the production of 1,3-PDO [5]. Several metabolic manipulations have been performed in natural producers to optimize the synthesis of 1,3-PDO. For example, in *K*. *pneumoniae* the reductive glycerol pathway has been enhanced, and the oxidative glycerol pathway or competing metabolite pathways have been eliminated, increasing 1,3-PDO yields [84]. Another strategy involves the manipulation of cofactor availability. In *K*. *oxytoca*, this has been achieved by introducing a formate dehydrogenase from *Candida boidinii* to regenerate NADH [89]. On the other hand, manipulation of redox metabolism in *E*. *coli* can be achieved using mutations redox regulators, such as ArcA, to enhance the synthesis of reduced metabolites [91]. This strategy has also been applied to increase the availability of reducing equivalents for the synthesis of 1,3-PDO in *E*. *coli* recombinants containing multiple genetic modifications [92].

## RECOMBINANT PROTEIN PRODUCTION

Several *E*. *coli* strains have been examined as potential MCFs to produce recombinant proteins at high titres [93], most often in high-cell-density cultures. A comprehensive overview of the main *E*. *coli* strains used in recombinant protein production processes and their characteristics has been recently published by Waegeman and Soetaert [94]. Although *E*. *coli* B and *E*. *coli* K12 are equally used as hosts for recombinant protein production (*ca*. 47% and 53%, respectively), *E*. *coli* BL21 is by far the most commonly used MCF (*ca*. 35%) in academia [95, 96]. This figure is probably even higher in industrial setups.

Acetate formation in *E*. *coli* cultures grown under either oxic or micro-oxic/anoxic conditions still represents a major problem in the industrial application of this microorganism [35, 97, 98]. As hinted before, acetate is mostly produced when respiratory pathways are saturated and NADH can no longer be re-oxidized [99]. This, in turn, causes a shunt in the main catabolic pathways by re-directing carbon precursors into acetate (the synthesis of which does not generate NADH, in contrast with the four molecules of NADH generated by each turn of the TCA cycle, Fig. 1), and thus allowing to achieve redox homeostasis. A plethora of different strategies have been devised to increase recombinant protein formation and to decrease acetate formation, including optimization of the bioprocess conditions as well as metabolic engineering of production hosts [100]. These attempts can be categorized in three main classes: [i] deleting genes involved in acetate formation, [ii] avoiding overflow metabolism by limiting D-glucose uptake *via* alteration of the degree of oxidation of the carbon source, applying alternative feeding strategies, or by engineering the D-glucose uptake system, and [iii] circumventing overflow metabolism by re-directing central metabolic fluxes and preserving sufficient precursors for amino acids synthesis, the actual building blocks of proteins.

Acetate has been postulated to be a potential signaling molecule in the activation of the Arc system [65], adding a further level of complexity to the ArcBA-mediated metabolic responses in *E*. *coli*. Vemuri *et al*. [101] reasoned that if cells are provided with an efficient mechanism to reduce high NADH/NAD^+^ ratios (that result in production of acetate) while preventing the Arc-dependent repression of the TCA cycle, additional carbon skeletons would be available for both biomass generation and recombinant protein production. The authors constructed an *E. coli arcA* mutant that overexpresses a NADH oxidase from *Streptococcus pneumoniae* [101]. Using steady-state, D-glucose − limited chemostat cultures, they were able to expose a strong correlation between acetate formation and the intracellular redox ratio [102]. Moreover, delay of acetate overflow in the engineered strain allowed to attain a titer of the model recombinant protein β-galactosidase *ca*. 2-fold higher than that of the wild-type strain.

Recent studies also allowed to explain a particular phenotype of the industrially-relevant *E*. *coli* BL21. By comparing the biomass yields and ^13^C-metabolic flux analysis of a Δ*arcA* Δ*iclR* double knock-out constructed in two genetic backgrounds (*E*. *coli* MG1655 and BL21) grown either under D-glucose-excess or D-glucose-limited conditions, it turned out that the metabolic similarity between both strains arise from mutations that affect both *arcA* expression and the promoter region of the gene encoding IclR, a local regulator which controls the transcription of the glyoxylate pathway genes within the *aceBAK* operon [103, 104]. These redox MCFs proved adequate for industrial purposes, as isocitrate was directly converted into succinate and malate, preventing carbon loss as CO_2_ and considerably diminishing the synthesis of acetate.

## OTHER METABOLIC ENGINEERING STRATEGIES FOR THE MANIPULATION OF THE REDOX HOMEOSTASIS

Although several strategies and approaches complementary to those discussed in this mini-review have been explored for the manipulation of the redox state in MCFs designed for reduced biochemicals production, some of them deserve special attention because of their relevance. A recent study by Bidart *et al*. [105] exploited the possibility of using partial deletions in the ArcB regulator in order to obtain graded phenotypes. This constituted a first-case study on the effects of confined deletions in a global regulator (as opposed to the elimination of the entire gene) for metabolic engineering endeavors. A gradual impact of these deletions on the distribution of metabolic fluxes was observed under anoxic growth conditions, and most of the changes could be traced to the redox state of the cells. The incremental differences observed both in the redox homeostasis and central carbon fluxes among the mutants make them potentially attractive MCFs for biotechnological purposes.

Other studies dealt with the direct manipulation of the redox state of the cell by stimulating high NAD(P)H/NAD(P)^+^ ratios. For instance, Zhou *et al*. [106] manipulated the transcriptional control of the genes encoding the components of the pyruvate dehydrogenase complex by using promoters of the *pfl* genes. Doubling of the reducing power output was achieved when D-glucose was converted into acetyl-CoA through the EMP pathway followed by oxidation of pyruvate by the anoxically-active pyruvate dehydrogenase complex (*i*.*e*., D-glucose → 2acetyl-CoA + 4NADH). Martínez *et al*. [107] constructed a recombinant *E*. *coli* strain by replacing *gapA*, encoding the native NAD^+^-dependent glyceraldehyde-3-phosphate dehydrogenase, with *gapC* from *Clostridium acetobutylicum*, which encodes a NADP^+^-dependent glyceraldehyde-3-phosphate dehydrogenase. The recombinant produced 2 moles of NADPH, instead of NADH, per mole of D-glucose consumed. The effectiveness of the NADPH enhancing system was analyzed in the recombinant MCFs by evaluating the production of lycopene and ɛ-caprolactone, the synthesis of which consumes NADPH, as model systems. The authors elegantly demonstrated that the synthesis of both heterologous molecules was favored in the recombinant MCFs when compared to the parental strain. Another interesting approach reported by Zhu *et al*. [108] showed that controlled respiration levels in *E*. *coli* can be exploited to allow wide operating windows in terms of O_2_ availability, thus allowing for the formation of reduced bioproducts even under fully oxic conditions. The authors added different amounts of coenzyme Q1 (an ubiquinone analogue) to oxic cultures of Δ*ubiCA* mutant strains (*i*.*e*., lacking the critical enzymes involved in ubiquinone synthesis). In this case, the target metabolite was ethanol, using glycerol as the carbon source. The recombinant MCF, carrying the *pdc* and *adhB* genes from *Zymomonas mobilis*, produced high ethanol titers under conditions in which the original strain was not able to synthesize any reduced by-product derived from the oxic glycerol catabolism.

## SUMMARY AND OUTLOOK

From the data presented in the preceding sections, it is clear that the relative lack of knowledge on the cellular wiring of regulatory networks under conditions relevant to both laboratory and industrial applications represents one of the most significant hurdles to be overcome for the efficient design of MCFs. However, a suite of different strategies that could offer interesting alternatives to tackle this issue has emerged in recent years.

In the first place, genome-scale and specific metabolic models are starting to play an important role in deciphering the cellular and metabolic characteristics of microbial cells *in silico* [109, 110]. The number of genome-scale metabolic models is rapidly increasing and their quality is also improving. Strategies devised to incorporate experimental data, such as high-throughput -omics data, have also been developed to enhance the quality and the accuracy of metabolic models. In close connection with these *in silico* approaches, synthetic biology is rapidly emerging as a relevant field for the rational manipulation of MCFs. Synthetic biology can be defined as the engineering of biology, *i*.*e*., the synthesis of complex, biologically-based (or inspired) systems, which could display functions that do not exist in nature. The possibility of developing an entirely synthetic host for efficient target-molecule production presents great opportunities for further research. In this sense, synthetic biology can significantly advance metabolic engineering by both contributing tools (*e*.*g*., minimal genome hosts, vectors, genetic controllers, and characterized enzymes), and by aiding potential interventions to metabolism at one or many of the following levels: [i] enhancement in the rate of substrate uptake, [ii] reduction of flux(es) to undesirable by-products, and enhancement of precursors and cofactor availability, [iii] introduction of relevant heterologous pathway(s) and optimization of the activity of its constituent enzymes, and [iv] export of the product to the extracellular medium in order to shift the concentration equilibrium towards product formation.

This exciting and extremely dynamic scenario will certainly lead to the development of better strategies to manipulate central and peripheral pathways to enhance the production of reduced biochemicals and other molecules of industrial interest.

## Supplementary Material

Genes of *E. coli* encoding metabolic-related functions regulated by the ArcBA and CreBC two-component systems. Data were compiled from Lynch and Lin [1] and Salmon et al. [2], and the specific references cited below.

## Abbreviations

MCF, microbial cell factory; PHAs, polyhydroxyalkanoates; PHB, poly(3-hydroxybutyrate); 1,3-PDO, 1,3-propanediol; EMP, Embden-Meyerhof-Parnas; PP, pentose phosphate; TCA cycle, tricarboxylic acid cycle; CoA, coenzyme A; PFL, pyruvate-formate lyase
