# Supplementary material for: Escherichia coli redox mutants as microbial cell factories for the synthesis of reduced biochemicals
Source: Comput Struct Biotechnol J. 2013 Jan 18;3:e201210019. doi: 10.5936/csbj.201210019 (PMC3962086; doi:10.5936/csbj.201210019)
Supplement: Genes of E. coli encoding metabolic-related functions regulated by the ArcBA and CreBC two-component systems. Data were compiled from Lynch and Lin [1] and Salmon et al. [2], and the specific references cited below. [file CSBJ-3-e201210019_SM0001.pdf]

Table S1. Genes of *E. coli* encoding metabolic-related functions regulated by the ArcBA and CreBC two-component systems. Data were compiled from Lynch and Lin [1] and Salmon et al. [2], and the specific references cited below.

ArcBA system			
Gene(s)	Function(s)	Regulation	Ref.
aceBA	Isocitrate lyase (AceA); catalyzes the cleavage of isocitrate or the condensation of glyoxylate and succinate. Malate synthase A (AceB); converts acetyl-CoA and glyoxylate into malate and CoA	–	[3]
acnA, *acnB*	Aconitase; catalyzes the reversible isomerization of citrate and iso-citrate *via cis*-aconitate in the TCA cycle	–	[4]
cydAB	Cytochrome *d* oxidase; catalyzes the oxidation of ubiquinol-8 during oxic respiration as O_2 becomes limiting	+	[5,6]
cyoABCDE	Cytochrome *o* oxidase; catalyzes the oxidation of ubiquinol-8 during oxic respiration under O_2-rich growth conditions	–	[5]
fumAC	Fumarase; interconverts fumarate and L-malate in the TCA cycle, and participates in the reductive pathway from oxaloacetate to succinate during anoxic growth	+	[7]
glcDEF	Glycolate oxidase; converts glycolate into glyoxylate in the glyoxylate shunt	–	[6]
gltA	Citrate synthase; catalyzes the condensation of acetyl-CoA and oxaloacetate to form citrate	–	[8]
icd	Isocitrate dehydrogenase; catalyzes the conversion of isocitrate to α-ketoglutarate, thereby forming NADH and CO_2	–	[6]
lldPRD	L-Lactate permease, regulator, and dehydrogenase, which interconvert pyruvate and L-lactate	–	[6]
lpdA	Lipoamide dehydrogenase; a component of the pyruvate dehydro-genase complex, α-ketoglutarate dehydrogenase, and the glycine-cleavage multi-enzyme systems	–	[9]
mdh	Malate dehydrogenase; catalyzes the reversible oxidation of malate to oxaloacetate, thereby forming NADH	–	[6]
ndh	Respiratory NADH dehydrogenase II; a membrane-bound (but non-H^+–translocating) monomeric flavoprotein	+	[6,10]
pflB	Pyruvate-formate lyase; catabolizes pyruvate under anoxic conditions	+	[11,12]
ptsG	D-Glucose–specific permease of the sugar phosphotransferase system	–	[13]
sdhCDAB	Succinate dehydrogenase; oxidizes succinate to fumarate and transfers electrons to ubiquinone in the oxic respiratory chain	–	[6]
sucABCD	Succinyl-CoA synthase; catalyzes the interconversion of succinyl-CoA and succinate by means of GTP hydrolysis, both in the TCA cycle and the reductive branch	–	[14]
**CreBC system**			
Gene	Function(s)	Regulation	Ref.
pta-ackA	Phosphate acetyltransferase (Pta) catalyzes the conversion of acetyl-CoA into acetyl-*P*, whereas acetate kinase (AckA) converts acetyl-*P* into acetate, thereby forming ATP	+	[15]
talA	Transaldolase A; converts D-glyceraldehyde-3-*P* and D-sedoheptulose-7-*P* into D-fructose-6-*P* and D-erythrose-4-*P* in the non-oxidative branch of the PP pathway	+	[15]

References

1. Lynch AS, Lin ECC (1996) Responses to molecular oxygen. In: Neidhardt FC, Curtiss III R, Ingraham JL, Lin ECC, Low KB, et al., editors. *Escherichia coli* and *Salmonella*: cellular and molecular biology, vol. 1. Washington, D.C.: ASM Press. pp. 1526-1538.
2. Salmon KA, Hung SP, Steffen NR, Krupp R, Baldi P, et al. (2005) Global gene expression profiling in *Escherichia coli* K12: effects of oxygen availability and ArcA. J Biol Chem 280: 15084-15096.
3. Lorca GL, Ezersky A, Lunin VV, Walker JR, Altamentova S, et al. (2007) Glyoxylate and pyruvate are antagonistic effectors of the *Escherichia coli* IclR transcriptional regulator. J Biol Chem 282: 16476-16491.
4. Cunningham L, Gruer MJ, Guest JR (1997) Transcriptional regulation of the aconitase genes (*acnA* and *acnB*) of *Escherichia coli*. Microbiology 143: 3795-3805.
5. Cotter PA, Gunsalus RP (1992) Contribution of the *fnr* and *arcA* gene products in coordinate regulation of cytochrome o and d oxidase (*cyoABCDE* and *cydAB*) genes in *Escherichia coli*. FEMS Microbiol Lett 70: 31-36.
6. Liu X, De Wulf P (2004) Probing the ArcA-P modulon of *Escherichia coli* by whole genome transcriptional analysis and sequence recognition profiling. J Biol Chem 279: 12588-12597.
7. Park SJ, Gunsalus RP (1995) Oxygen, iron, carbon, and superoxide control of the fumarase *fumA* and *fumC* genes of *Escherichia coli*: role of the *arcA*, *fnr*, and *soxR* gene products. J Bacteriol 177: 6255-6262.
8. Park SJ, McCabe J, Turna J, Gunsalus RP (1994) Regulation of the citrate synthase (*gltA*) gene of *Escherichia coli* in response to anaerobiosis and carbon supply: role of the *arcA* gene product. J Bacteriol 176: 5086-5092.
9. Li M, Ho PY, Yao S, Shimizu K (2006) Effect of *lpdA* gene knockout on the metabolism in *Escherichia coli* based on enzyme activities, intracellular metabolite concentrations and metabolic flux analysis by ^{13}C-labeling experiments. J Biotechnol 122: 254-266.
10. Green J, Anjum MF, Guest JR (1997) Regulation of the *ndh* gene of *Escherichia coli* by integration host factor and a novel regulator, Arr. Microbiology 143: 2865-2875.
11. Sawers G (1993) Specific transcriptional requirements for positive regulation of the anaerobically inducible *pfl* operon by ArcA and FNR. Mol Microbiol 10: 737-747.
12. Drapal N, Sawers G (1995) Promoter 7 of the Escherichia coli *pfl* operon is a major determinant in the anaerobic regulation of expression by ArcA. J Bacteriol 177: 5338-5341.
13. Shin D, Cho N, Kim YJ, Seok YJ, Ryu S (2008) Up-regulation of the cellular level of *Escherichia coli* PTS components by stabilizing reduced transcripts of the genes in response to the low oxygen level. Biochem Biophys Res Commun 370: 609-612.
14. Park SJ, Chao G, Gunsalus RP (1997) Aerobic regulation of the *sucABCD* genes of *Escherichia coli*, which encode α-ketoglutarate dehydrogenase and succinyl coenzyme A synthetase: roles of ArcA, Fnr, and the upstream *sdhCDAB* promoter. J Bacteriol 179: 4138-4142.
15. Avison MB, Horton RE, Walsh TR, Bennett PM (2001) *Escherichia coli* CreBC is a global regulator of gene expression that responds to growth in minimal media. J Biol Chem 276: 26955-26961.
